# Global ^13^C tracing and metabolic flux analysis of intact human liver tissue ex vivo

**DOI:** 10.1038/s42255-024-01119-3

**Published:** 2024-08-29

**Authors:** Nina Grankvist, Cecilia Jönsson, Karin Hedin, Nicolas Sundqvist, Per Sandström, Bergthor Björnsson, Arjana Begzati, Evgeniya Mickols, Per Artursson, Mohit Jain, Gunnar Cedersund, Roland Nilsson

**Affiliations:** 1 Cardiovascular Medicine Unit, Department of Medicine Solna, Karolinska Institutet, Stockholm, Sweden; 2 Division of Cardiovascular Medicine, Karolinska University Hospital, Stockholm, Sweden; 3 Center for Molecular Medicine, Karolinska Institutet, Stockholm, Sweden; 4 Department of Health, Medicine and Caring Sciences, Linköping University, Linköping, Sweden; 5 Department of Biomedical engineering, Linköping University, Linköping, Sweden; 6 Department of Surgery, Linköping University Hospital, Linköping, Sweden; 7 Department of Biomedical and Clinical Sciences, Linköping University, Linköping, Sweden; 8 Department of Medicine & Pharmacology, University of California, San Diego, La Jolla, CA USA; 9 Department of Pharmacy, Uppsala University, Uppsala, Sweden; 10 Sapient Bioanalytics, San Diego, CA USA

**Keywords:** Metabolomics, Metabolic syndrome, Biochemical reaction networks, Translational research, Hepatocytes

## Abstract

Liver metabolism is central to human physiology and influences the pathogenesis of common metabolic diseases. Yet, our understanding of human liver metabolism remains incomplete, with much of current knowledge based on animal or cell culture models that do not fully recapitulate human physiology. Here, we perform in-depth measurement of metabolism in intact human liver tissue ex vivo using global ^13^C tracing, non-targeted mass spectrometry and model-based metabolic flux analysis. Isotope tracing allowed qualitative assessment of a wide range of metabolic pathways within a single experiment, confirming well-known features of liver metabolism but also revealing unexpected metabolic activities such as de novo creatine synthesis and branched-chain amino acid transamination, where human liver appears to differ from rodent models. Glucose production ex vivo correlated with donor plasma glucose, suggesting that cultured liver tissue retains individual metabolic phenotypes, and could be suppressed by postprandial levels of nutrients and insulin, and also by pharmacological inhibition of glycogen utilization. Isotope tracing ex vivo allows measuring human liver metabolism with great depth and resolution in an experimentally tractable system.

## Abstract (web summary)

Grankvist et al. combine global isotopic tracing and metabolic flux modelling to characterize metabolic networks in human intact liver tissue.

## Main

The liver is the metabolic hub of the human body, responsible for central metabolic processes such as glucose storage and synthesis, lipoprotein production, nitrogen disposal and detoxification of harmful substances from the diet. Consequently, the liver plays a central role in the pathology of common metabolic disorders such as obesity, cardiovascular disease and diabetes. Metabolic dysfunction-associated steatotic liver disease (formerly non-alcoholic fatty liver disease) is a common feature of these disorders, which can trigger liver inflammation and lead to advanced liver disease, through mechanisms that still are not fully understood^1^. A solid understanding of human liver metabolism is, therefore, critical for elucidating the pathology of common metabolic disease.

Despite decades of study, our knowledge of human liver metabolism remains incomplete, in part due to the difficulty of measuring metabolic fluxes in the liver. Arteriovenous concentration differences can be used to estimate metabolite uptake and release fluxes across tissue beds^2,3^, but can be problematic in the liver due to complex vascularization where the portal vein is not easily accessible. Non-invasive isotope tracing with infusion of labelled nutrients followed by measurement of plasma metabolites has been the main technique for studying liver metabolism in humans in vivo. For example, ^13^C tracing has been used to determine differences in hepatic metabolism of fructose and glucose^4^, and deuterated water (^2^H_2_O) has been used to probe de novo lipogenesis^5^ by measuring ^2^H incorporation into blood lipids. While such in vivo measurements represent the gold standard for physiological relevance and have been invaluable in mapping out human liver metabolism, the information obtained in any single experiment is limited since metabolites of interest are not always present in plasma. Moreover, results can be difficult to interpret due to the complexity of whole-body metabolism, with contributions from multiple tissues and inter-organ metabolic cycles^6^. For example, while glucose has long been considered the primary substrate for lipogenesis in the liver^7^, recent studies that account for metabolic cycling instead point to lactate and acetate as the main substrates^8^. In addition, isotope tracing in humans in vivo requires large quantities of expensive isotopic tracers, rendering them difficult to scale up to larger cohorts.

While in vivo tracing studies have traditionally focused on hand-crafted analyses of specific pathways of interest, modern mass spectrometry and computational techniques now enable a more systematic analysis of human metabolism. Perhaps half of all metabolic enzymes encoded in the human genome are still uncharacterized^9^, and non-targeted isotope tracing provides opportunities to identify novel human metabolites and pathways on a large scale^10,11^. Moreover, metabolic flux analysis (MFA) methods developed in the biotechnology community allow interpretation of ^13^C tracing data in the context of a given metabolic network model to provide quantitative estimates of pathway activity^12,13^. These techniques yield rich information, but generally require measuring intracellular metabolites to obtain enough information for precise flux estimates. To date, modern ^13^C tracing and MFA methods have been applied to analyse liver metabolites mainly in rodents, using isolated hepatocytes^14,15^, liver perfusion^16^ or in vivo tracing^17^. While such animal studies are powerful, human metabolism differs markedly from that of rodents, with distinct lipoprotein profiles and bile acid metabolism^18,19^, several-fold higher basal metabolic rate^20^ and 10–15 times faster glucose turnover^21^. Direct measurement of human liver metabolism therefore remains crucial for translating findings from model organisms.

Here, we approach the problem of measuring liver metabolism by applying ^13^C isotope tracing and MFA to human liver tissue slices cultured ex vivo. Liver tissue cultures have been used for decades, in early biochemistry studies to elucidate biochemical pathways, and more recently for drug testing and fibrosis research^22–24^. While the technique has its caveats, including stress caused by tissue sectioning and challenges with ensuring good oxygen and nutrient perfusion, liver tissue can be maintained in good condition ex vivo for several days^22,25^. The approach preserves the full complement of liver cell types in their natural microenvironment and allows for easy experimental manipulation. We therefore reasoned that tissue cultures could be an excellent choice for in-depth analysis of human liver metabolism.

## Results

### Human liver cultured ex vivo retains metabolic function

**Fig. 1:**
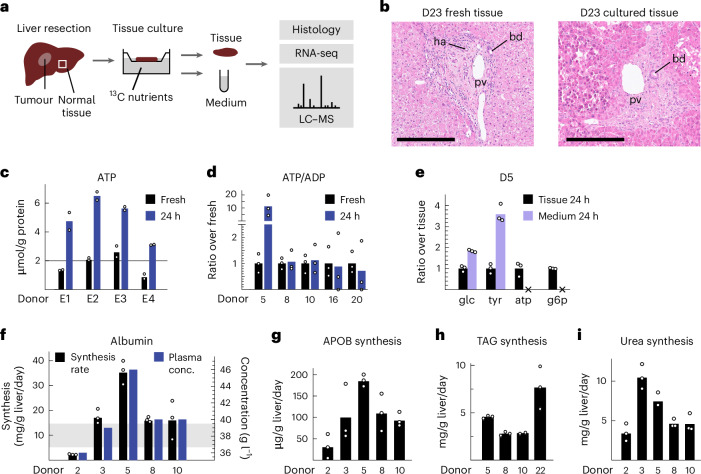
Cultured human liver retains metabolic function. **a**, Schematic of liver tissue sampling, culture and analysis. RNA-seq, RNA sequencing. **b**, Representative histology images of freshly resected (left) and 24-h cultured human liver tissue (right), from a total of 12 images. pv, portal vein; ha, hepatic artery; bd, bile duct. Scale bars, 250 µm. **c**, ATP content of freshly resected and 24-h cultured liver slices. **d**, ATP/ADP content, as in **c**. **e**, relative abundance of indicated metabolites in liver tissue and conditioned medium. g6p, glucose-6-phosphatase. **f**, Liver tissue synthesis rate (left scale) and plasma concentration (right scale) of albumin. Shaded area indicates the range of in vivo synthesis rate from previous reports (Supplementary Table 4). **g**, Synthesis rate of APOB. **h**, Synthesis rate of triacylglycerides (TAGs). **i**, Synthesis rate of urea. Data from *n* = 3 independent tissue slices are shown for each donor in **c**–**i**. Source data

**Extended Data Fig. 1:**
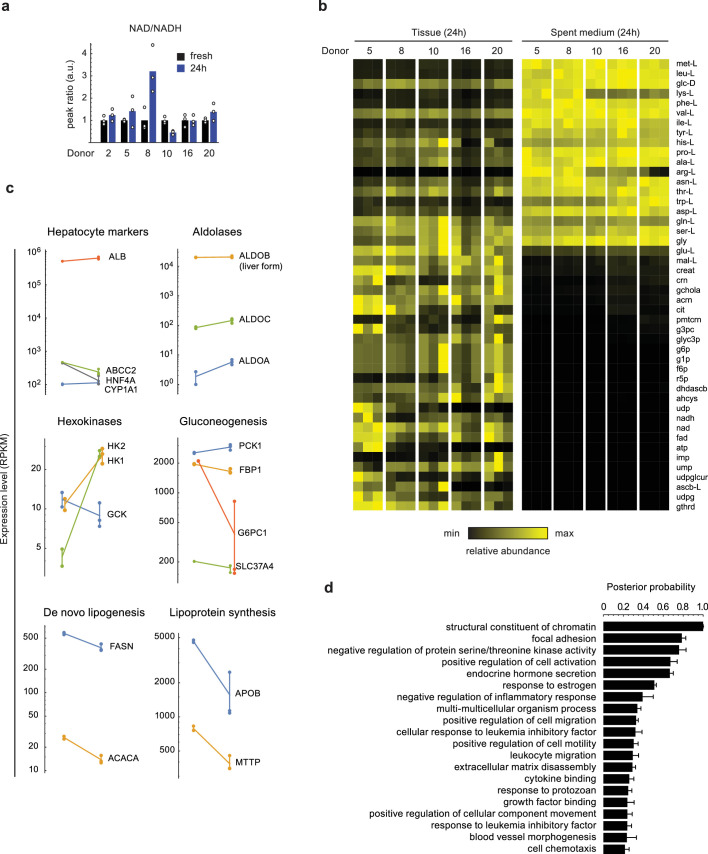
Metabolic state of culture liver slices. **a**, Relative NAD/NADH ratios in fresh liver tissue and cultured liver slices, normalized to fresh tissue ratio for each donor. **b**, Relative abundances of metabolites in cultured liver tissue and spent medium, normalized by maximum for each row. **c**, Expression of selected genes in freshly resected liver tissue and cultured liver slices, measured by RNA sequencing. Individual data points are shown, with vertical lines indicating the range of variation for clarity. **d**, GeneOntology enrichment analysis of mRNAs with increased expression in cultured liver slices vs. fresh tissue. Error bars indicate standard error of the posterior probability, as estimated by the MGSA R package. Data from n = 3 independent tissue slices are shown for each donor in a–c. Source data

We first sought to establish that intact human liver tissue cultured ex vivo retains key physiological and metabolic functions. While liver tissue has successfully been maintained in culture for several days^22^, we chose to perform experiments within 24 h after liver resection to minimize disturbances. Normal liver tissue was obtained from individuals undergoing surgery for resection of liver tumours (Supplementary Table 1). Individuals were fasted overnight, but received oral carbohydrates and were likely not depleted of glycogen. After resection, liver tissue was immediately sectioned into 150–250-μm slices, and cultured on membrane inserts (Fig. 1a) in a medium with nutrient levels approximating those in fasted-state plasma (Supplementary Table 2). This set-up has previously been shown to provide ample oxygenation and maintain tissue in excellent condition^25^. After 24 h of culture, tissue appeared anatomically intact with hallmark liver structures such as portal veins and bile ducts clearly visible (Fig. 1b). In high-quality slices, cell viability was above 90%, and liver tissue was in all cases free of cancer cells (Supplementary Table 3). ATP content was low in freshly sectioned, cold tissue as expected^25^, but increased in cultured slices to ~5 µmol per gram of protein (Fig. 1c), indicating metabolic viability. The ATP/ADP ratio was also well maintained in cultured slices (Fig. 1d), as was the NAD/NADH ratio (Extended Data Fig. 1a), indicating that energy charge and redox balance are preserved. In addition, metabolites that are normally intracellular, such as nucleotides and phosphorylated sugars, were absent from culture media (Fig. 1e and Extended Data Fig. 1b), indicating intact cell membranes. Cultured liver slices generally retained expression of hepatocyte markers and liver-specific enzymes, although genes involved in de novo lipogenesis and lipoprotein synthesis decreased (Extended Data Fig. 1c), possibly because insulin was absent in the baseline culture conditions. Gene-set enrichment analysis revealed expression signatures of wound healing processes and immune cell activation, as previously reported in sectioned liver tissue^26^, but not apoptosis pathways (Extended Data Fig. 1d).

To assess physiological liver function, we quantified synthesis and release of key liver products during the 24-h culture period. Liver slices synthesized albumin at a rate of 10–30 mg per gram of liver per day, comparable to previously reported in vivo rates (Fig. 1f and Supplementary Table 4)^27–29^. Remarkably, albumin production in cultured tissue closely reflected albumin levels in donor plasma (Fig. 1f), suggesting that individual differences in albumin homeostasis are maintained ex vivo. As an assessment of very-low-density lipoprotein (VLDL) synthesis rate, we measured apolipoprotein B (APOB) secretion rates of around 50–200 µg per gram of liver per day (Fig. 1g), somewhat lower than reported in vivo rates of around 200–400 µg per gram of liver per day in fasted individuals (Supplementary Table 4). If the measured APOB truly reflects VLDL particles, we would expect 2–8 mg triglyceride per gram of liver per day, based on VLDL composition^30^. Indeed, we observed triglyceride release rates in this range (Fig. 1h), indicating that liver slices produce mature VLDL particles. Liver slices formed 5–10 mg urea per gram per day (Fig. 1i), comparable with in vivo measurements around 20 mg per gram per day (Supplementary Table 4). Hence, the urea cycle is fully operational. Taken together, these data indicate that human liver ex vivo maintains metabolic functions at levels comparable to in vivo conditions.

### Mapping human liver metabolism with global ^13^C tracing

**Fig. 2:**
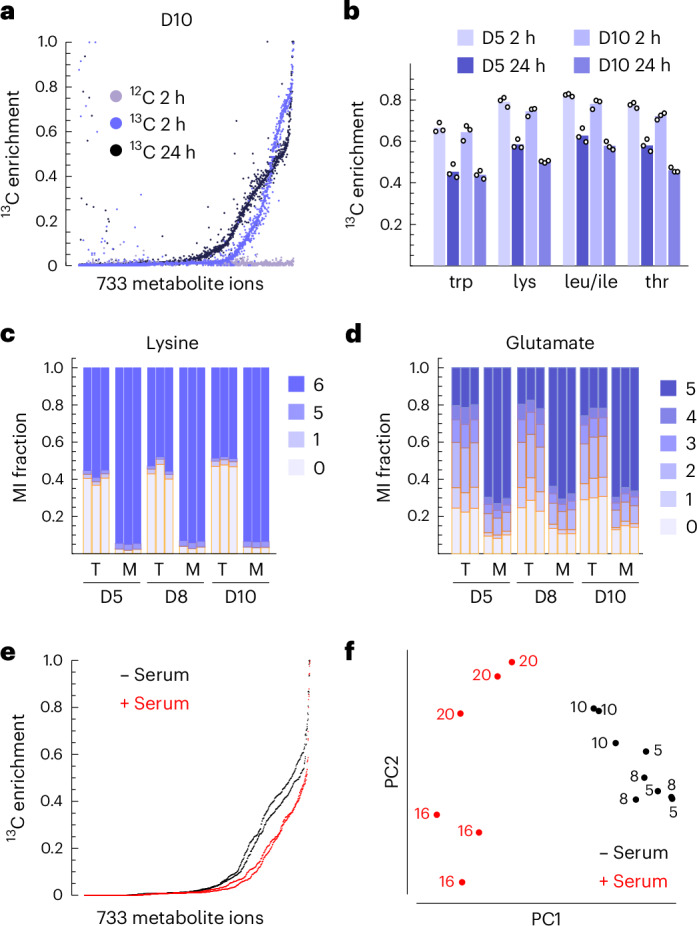
Global ^13^C tracing in human liver tissue. **a**, Distribution of ^13^C enrichment across putative metabolites in indicated conditions. Dots represent values for individual metabolites, from three tissue slices per condition. **b**, ^13^C enrichment in tissue slices for the indicated AAs. **c**, Mass isotopomer (MI) distribution of lysine in tissue (T) and medium (M). **d**, MI distribution of glutamate, as in **c**. **e**, Distribution of ^13^C enrichment in tissues cultured with (+) and without (−) human serum. Dots indicate the median of three slices per donor for each metabolite. **f**, The first two principal components (PCs) of ^13^C enrichment for 733 metabolites. Dots indicate individual liver slices; numbers indicate donors. Data from *n* = 3 independent tissue slices are shown for each donor in **a**–**d** and **f**. Source data

**Extended Data Fig. 2:**
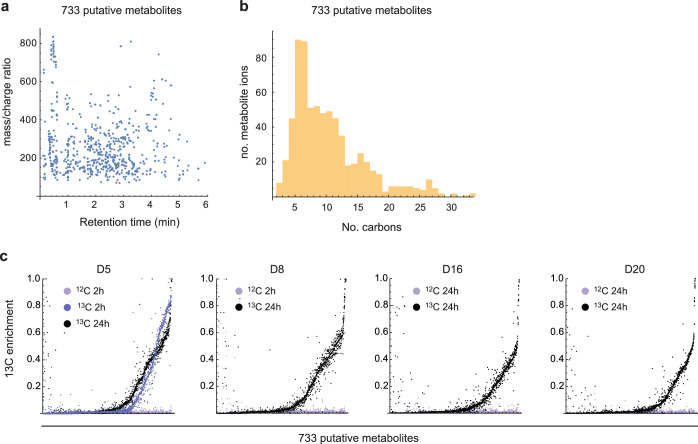
Properties of global isotope tracing data. **a**, Mass-charge ratio and retention time of 733 ions representing putative metabolites. **b**, Distribution of carbon number for all putative metabolites. **c**, ^13^C enrichment distribution in tissues after 2 h or 24 h labeling, and unlabeled (^12^C) controls. Dots represent values for individual metabolites, from three tissue slices per condition. Source data

To qualitatively assess metabolic activities in liver tissues in an unbiased manner, we performed ^13^C isotope tracing with a highly ^13^C-enriched medium in which all 20 amino acids (AAs) plus glucose were fully labelled with ^13^C. This design allows monitoring ^13^C incorporation into a wide variety of cellular products and metabolic intermediates in a single experiment^10^. Liquid chromatography–mass spectrometry (LC–MS) analysis of polar metabolites in ^13^C-labelled liver tissues and spent medium resulted in 733 LC–MS peaks representing putative metabolites (Extended Data Fig. 2a,b and Supplementary Data 1). About one-third of these metabolites gained detectable ^13^C enrichment after 2 h of labelling, increasing to nearly half at 24 h (Fig. 2a and Extended Data Fig. 2c), representing metabolites synthesized by the tissue during this time period. Essential AAs in tissues reached 60–80% ^13^C enrichment at 2 h (Fig. 2b), indicating good nutrient perfusion throughout the tissue. Assuming first-order kinetics, we expected that essential AAs in tissues would reach 100% ^13^C at ~6 h; however, we consistently observed lower ^13^C enrichment at 24 h (Fig. 2b). This suggests that a sizable fraction of liver AAs derive from breakdown of unlabelled (^12^C) tissue protein during this time period. Indeed, the liver is remarkable in its capacity for protein remodelling, with protein turnover in vivo reaching as high as 25% per day^31^. Interestingly, both essential and non-essential AA mass isotopomer distributions (MIDs) differed markedly between medium and tissue (Fig. 2c,d), indicating that a substantial pool of AAs does not freely exchange with the medium. Since cytosolic AAs generally exchange rapidly across the plasma membrane^32^, a possibility is that human liver sequesters AAs in lysosomes, as previously observed in rat liver^33^, and consistent with the discovery that AA sensing occurs in lysosomes^34^.

One caveat with measuring metabolism in cultured liver tissue is that typical culture medium is serum-free and, therefore, entirely lacks protein and fat (lipoproteins) as well as albumin-bound fatty acids. To explore the contribution of macromolecules to liver metabolism, we performed ^13^C tracing in medium supplemented with 50% dialysed human serum. Addition of dialysed serum did not alter medium metabolite concentrations markedly, but provided fatty acids and insulin at fasting levels (Supplementary Table 5), and should therefore more closely approximate the in vivo environment. We consistently observed lower ^13^C enrichment in serum-containing cultures (Fig. 2e). While the effect on individual metabolites was modest, cluster analysis of the full ^13^C enrichment profiles across all metabolites clearly distinguished serum-supplemented cultures, and also generally separated cultures by donor (Fig. 2f). These data suggest that liver slices metabolize serum-derived ^12^C nutrients, although isotope dilution of ^13^C tracers by serum-derived ^12^C metabolites (Supplementary Table 6) may also partly explain the effect.

**Fig. 3:**
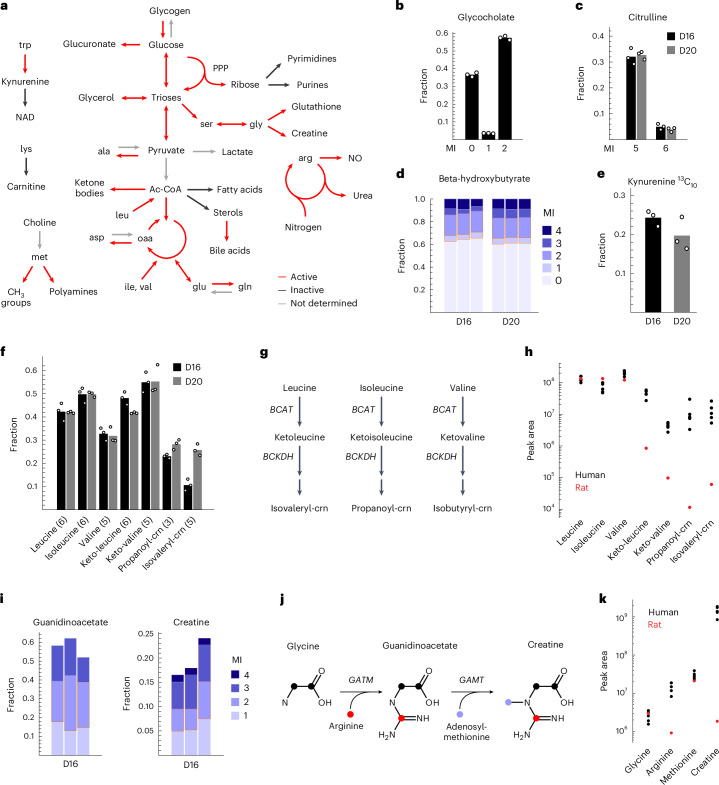
Systematic assessment of liver metabolic activities. **a**, Schematic indicating liver pathways whose activity in tissue slices could be inferred from untargeted ^13^C analysis. NAD, nicotinamide adenine dinucleotide; PPP, pentose phosphate pathway; Ac-CoA, acetyl-coenzyme A; NO, nitric oxide. **b**, Mass isotopomer (MI) fractions of glycocholate. **c**, MI fractions of citrulline. **d**, MI fractions of beta-hydroxybutyrate. **e**, ^13^C_10_ MI fraction of kynurenine. **f**, MI fraction of indicated BCAA metabolites, with MI number in parentheses. crn, carnitine. **g**, Simplified schematic of BCAA catabolism pathways. **h**, LC–MS peak areas of indicated metabolites in human liver from five donors (black) and in rat liver (red). Mean values across three tissue slices are shown. **i**, MI fractions of guanidinoacetate and creatine. **j**, Schematic of the creatine synthesis pathway; colour indicates origin of atoms. **k**, LC–MS peak areas of indicated metabolites, as in **h**. Data from *n* = 3 independent tissue slices are shown for each donor in **b**–**f** and **i**. Source data

**Extended Data Fig. 3:**
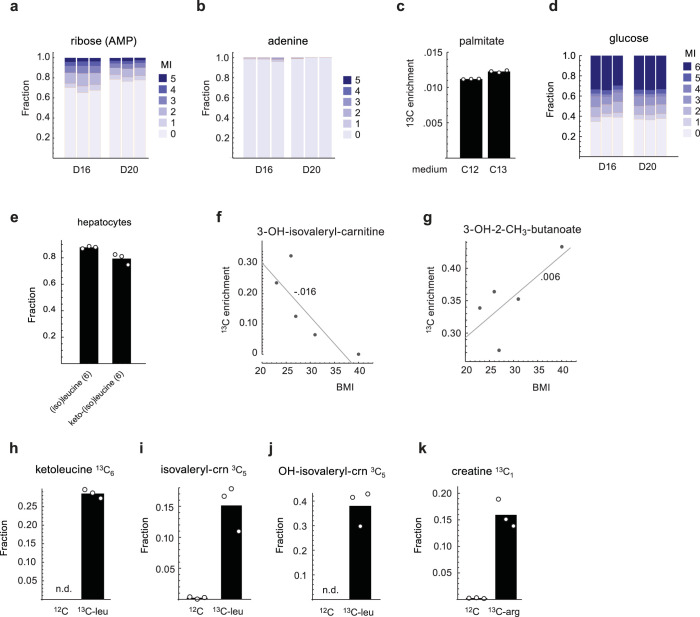
Isotope labeling of reporter metabolites and branched-chain amino acid products. **a**, Liver tissue MI fractions of the ribose moiety of AMP, deconvoluted. **b**, MI fractions of adenine. **c**, ^13^C enrichment in liver tissue palmitate, from cultures with ^12^C (unlabeled) and ^13^C (deep labeling) medium. **d**, MI fractions in glucose. **e**, MI fraction of indicated branched-chain amino acid (BCAA) metabolites, with mass isotopomer number in parentheses, in human hepatocytes. **f-g**, Liver tissue ^13^C enrichment in branched-chain AA metabolites 3-OH-isovaleryl-carnitine (f) and 2-OH-3-CH_3_-butanoate (g) *vs*. donor BMI. **h**–**j**, Indicated MI fractions of ketoleucine (h), isovaleryl-carnitine (i) and hydroxy-isovaleryl-carnitine (j) in liver tissue incubated with U-^13^C-leucine or unlabeled (^12^C) control. **k**, ^13^C_1_ MI fraction of creatine in liver tissue incubated with U-^13^C-arginine or unlabeled (^12^C) control, corrected for naturally occurring ^13^C. Data from n = 3 independent tissue slices are shown for each donor in a–e and h–k. Source data

To systematically assess metabolic activity in liver tissues, we analysed ^13^C MIDs of ‘reporter’ metabolites that qualitatively indicate activity of specific pathways (Fig. 3a and Supplementary Table 7). Overall, we observed that canonical liver pathways remain functional ex vivo. For example, bile acid synthesis from pre-existing sterols was readily observable as ^13^C_2_ glycine incorporation into glycocholate (Fig. 3b); urea cycle activity was evident from ^13^C_5_ citrulline, with a smaller amount of ^13^C_6_ citrulline indicating nitric oxide synthase activity (Fig. 3c); and formation of ^13^C beta-hydroxybutyrate revealed ongoing ketone body synthesis (Fig. 3d). The kynurenine pathway, which provides precursors for NAD synthesis and mainly occurs in liver^35^, was also readily measurable (Fig. 3e). Purine nucleotides showed ^13^C enrichment in the ribose moiety (Extended Data Fig. 3a), while the nucleobase was unlabelled (Extended Data Fig. 3b), indicating ongoing ribose synthesis and nucleotide salvage, but little de novo synthesis of purines and pyrimidines, as would be expected in tissues with little cell proliferation. We did not observe de novo synthesis of fatty acids (Extended Data Fig. 3c), possibly due to lack of insulin simulation in this baseline condition. Liver tissue glucose exhibited a complex MID (Extended Data Fig. 3d) consisting of about one-third ^13^C_6_, indicating uptake from medium, and one-third ^13^C_0_ (unlabelled), which likely derives from glycogen breakdown; the remainder (^13^C_1_ to ^13^C_5_) might reflect gluconeogenesis or pentose phosphate pathway activity.

While most of the results from the ^13^C analysis were expected, some surprising observations stood out. We noticed substantial ^13^C labelling in several branched-chain amino acid (BCAA) catabolites (Fig. 3f), including the corresponding keto acids and branched-chain acylcarnitines, indicating that BCAAs are transaminated and oxidized by liver tissue (Fig. 3g). Since such BCAA activity could derive from non-parenchymal cells, we also performed ^13^C tracing in isolated human hepatocytes. We again observed ^13^C labelling of branched-chain keto acids (Extended Data Fig. 3e), suggesting that at least a part of the observed BCAA activity occurs in hepatocytes. Interestingly, for two BCAA derivatives, ^13^C enrichment also correlated with donor body mass index (Extended Data Fig. 3f,g), suggesting that their formation might be related to individual metabolic phenotypes. Follow-up tracing experiments with ^13^C-labelled leucine confirmed labelling of these BCAA catabolites (Extended Data Fig. 3h–j). Although appearance of the isotopic label in keto acids can arise simply due to exchange flux through branched-chain amino acid transferase (BCAT), the observed ^13^C labelling of branched-chain acylcarnitines does indicate net flux in the catabolic direction, since the dehydrogenase step can be assumed to be irreversible. Elevated BCAA in plasma can cause insulin resistance and is predictive of future diabetes, but the underlying mechanisms remain elusive^36^. Liver is generally considered to lack the BCAA transaminase (BCAT; Fig. 3g); instead, transamination is thought to occur largely in muscle, which releases the corresponding keto acids for oxidation in the liver^36^. In contrast, our data indicate that BCAT is active in the human liver, enabling direct catabolism of BCAA, although the magnitude of this flux cannot be estimated from our data. The current consensus view appears to be based on studies in rat liver, which exhibits low BCAT levels^37^, while BCAT expression is higher in human liver^38^. BCAA catabolites in rat liver cultured ex vivo were much lower than in human liver (Fig. 3h), further supporting the notion that BCAA metabolism is different in humans.

We also found marked ^13^C labelling of guanidinoacetate and creatine (Fig. 3i), indicating that creatine was synthesized de novo from glycine and arginine (Fig. 3j), and this was confirmed using a ^13^C-arginine tracer (Extended Data Fig. 3k). In contrast, rat liver has been shown to lack the GATM enzyme that forms guanidinoacetate^39^, so that creatine synthesis must rely on import of guanidinoacetate synthesized by the kidneys^40^. However, guanidinoacetate synthesis in human kidneys does not appear sufficient to cover demand^41^, and *GATM* mRNA has been detected in several human tissues^42^. We observed that cultured human liver sustained much higher creatine levels than rat liver (Fig. 3k). Therefore, our data support the view that the complete creatine synthesis pathway is present in the human liver.

### Quantifying metabolic fluxes in liver tissue

**Fig. 4:**
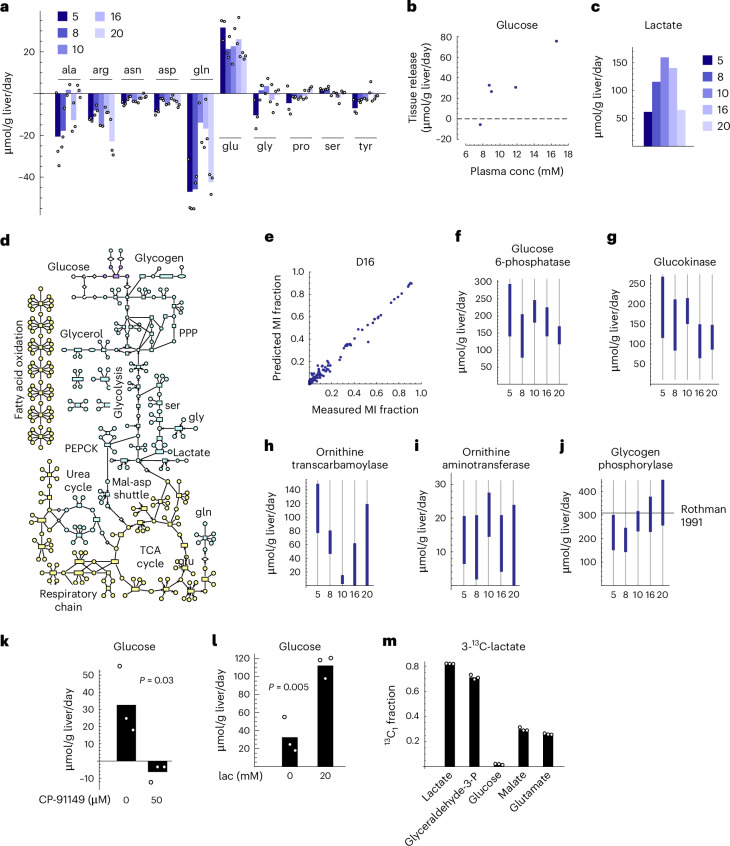
Metabolic flux analysis in liver tissue. **a**, Uptake (negative) and release (positive) fluxes for indicated metabolites from culture medium. Numbers indicate individual donors. **b**, Glucose release flux versus donor plasma glucose concentration. **c**, Lactate release flux. **d**, Schematic of the model used for ^13^C MFA, with pathways and compartments indicated. Blue, cytosol; red, mitochondrion; green, endoplasmic reticulum. **e**, Model-predicted versus measured MI fractions for all included metabolites. **f**–**j**, 90% confidence intervals for flux through glucose-6-phosphatase (**f**), glucokinase (**g**), ornithine transcarbamoylase (**h**), ornithine aminotransferase (**i**) and glycogen phosphorylase (**j**). Solid line in **j** indicates literature values from source publications listed in Supplementary Table 4. **k**, Glucose release flux in liver tissue with and without the glycogen phosphorylase inhibitor CP-91149. **l**, Glucose release flux in liver tissue incubated with indicated concentrations of lactate (lac). **m**, ^13^C_1_ MI fractions of indicated metabolites in liver slices incubated with 20 mM 3-^13^C-lactate. Confidence intervals in **f**–**j** were obtained by the profile likelihood method (Methods) based on *n* = 3 independent tissue slices. *P* values were calculated using Student’s two-sided *t*-test from *n* = 3 tissue slices. Source data

**Extended Data Fig. 4:**
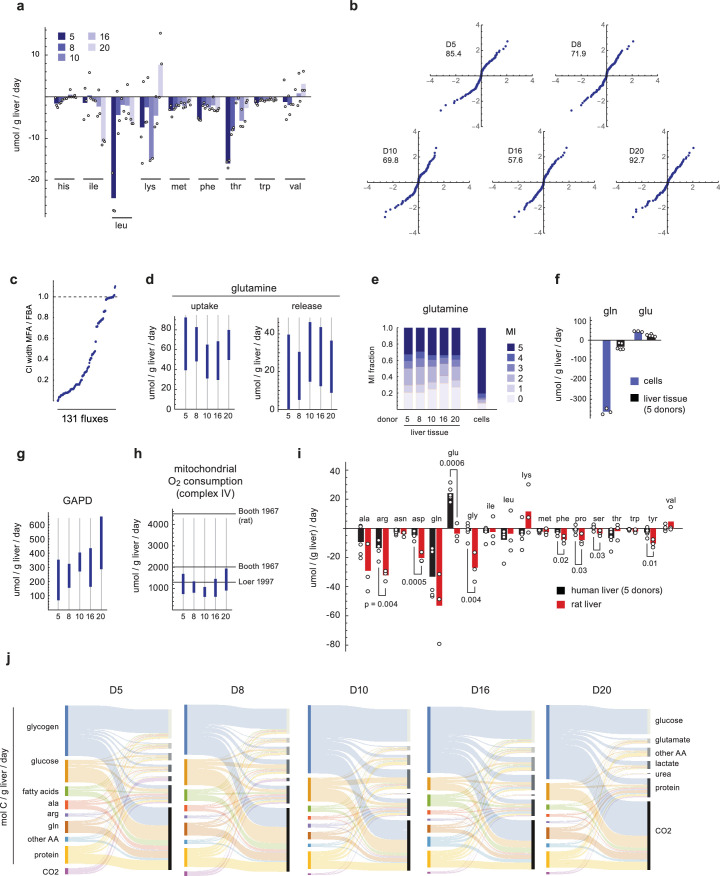
Uptake/release rates and metabolic fluxes in liver slices. **a**, Uptake (negative) and release (positive) rates of essential amino acids in liver tissue. Numbers indicate individual donors. **b**, Quantile-quantile plots of 149 residual MI errors from model fitting. Numbers in top left quadrants indicate χ^2^ statistic for each fitted model. **c**, Comparison of confidence intervals (CIs) for 131 fluxes from models fitted with (MFA) and without (FBA) mass isotopomer information. Ratio of confidence interval width is shown. **d**, 90% confidence intervals for glutamine uptake and release flux. **e**, MIDs of glutamine in liver tissue from indicated donors (left) and in hepatocytes (right). **f**, uptake/release of glutamine (gln) and glutamate (glu) in human tissue and hepatocytes. **g**–**h**, 90% confidence intervals for flux through glycerol-3-phosphate dehydrogenase (g) and mitochondrial complex IV (h). Solid lines in (h) indicate literature values from source publications listed in Supplementary Table 3. **i**, uptake/release of indicated amino acids in human liver (black) and rat liver (red). Dots indicate mean value per donor for human samples, and individual tissue slices for rat. p-values are indicated where p < 0.01, calculated using Student’s two-sided t-test from n = 3 rat tissue slices vs. n = 5 human liver donors. **j**, Sankey diagram of carbon flow from substrates (left) to products (right) in liver tissue from indicated donors. Confidence intervals in d,g,h were obtained by the profile likelihood method (see Methods) based on n = 3 independent tissue slices. Source data

While ^13^C labelling yields qualitative information on pathway activity, a thorough understanding of liver metabolism requires quantitative data on metabolic fluxes. We first quantified net uptake and release rates of major substrates and products (Fig. 4a). Among non-essential AAs, liver tissue mainly consumed alanine, arginine and glutamine (Fig. 4a). Tissues also released substantial amounts of glutamate, possibly from deamination of glutamine, similar to recent arteriovenous data in pigs^3^. Essential AA uptake was generally small (Extended Data Fig. 4a), although rates varied between donors, possibly reflecting individual differences. Net production of glucose occurred in most cases (Fig. 4b), as expected in fasted-state liver. Importantly, glucose release correlated with donor plasma glucose concentration (Fig. 4b), suggesting that individual differences in hepatic glucose production are preserved in cultured liver tissues. Net release of lactate occurred in all cases (Fig. 4c).

To integrate uptake–release data with ^13^C MIDs and obtain quantitative estimates of intracellular fluxes, we performed model-based ^13^C MFA. We developed an atom-level metabolic network model for a subset of liver metabolic pathways focusing on carbohydrate and AA metabolism as well as protein synthesis and degradation (Fig. 4d, Supplementary Data 2 and Supplementary Note 1). For each donor, we fit this model to a dataset of 151 measured mass isotopomers and fluxes (Fig. 4e and Extended Data Fig. 4b). We achieved a statistically acceptable fit in all cases (Extended Data Fig. 4b), indicating that the model can explain all included data, and that all measurements are internally consistent. From the fitted metabolic network models, we obtained estimates of 144 intracellular net fluxes (Supplementary Table 8), providing a detailed view of the liver metabolic state. The majority of these estimates could not be obtained from label-free flux balance analysis (Extended Data Fig. 4c), demonstrating that ^13^C labelling is crucial for measuring metabolism in this system. For example, we observed simultaneous glucose synthesis by glucose-6-phosphatase (Fig. 4f) and catabolism via glucokinase (Fig. 4g), at rates markedly higher than the net glucose production. This phenomenon might reflect distinct metabolism in subpopulations of hepatocytes, given that glucose-6-phosphatase and glucokinase are known to be ‘zonated’, with preferential expression near the portal and central veins, respectively^43^. Similarly, glutamine uptake and release occurred simultaneously (Extended Data Fig. 4d), possibly reflecting presence of both glutaminase-expressing periportal hepatocytes and glutamine synthetase-expressing pericentral hepatocytes^44^. In contrast, ^13^C isotope tracing in primary human hepatocytes showed that glutamine uptake predominated over glutamine synthesis, as indicated by a high ^13^C_5_ isotopomer (Extended Data Fig. 4e) and comparatively high net glutamine uptake (Extended Data Fig. 4f). Because glutamine synthase is expressed in a small population of periportal hepatocytes^45^ and hepatocyte isolation shows batch-to-batch variation in subpopulations^46^, this difference could indicate incomplete representation of liver cell types in monolayer culture, or alternatively reflect higher exposure to medium in monolayer cultures. We also observed flux through both the urea cycle (Fig. 4h) and arginine catabolism to glutamate (Fig. 4i) in liver slices, indicating that arginine served both as nitrogen carrier and fuel.

We did not observe net gluconeogenesis from pyruvate, as net GAPDH flux was always in the oxidizing direction (Extended Data Fig. 4g). Instead, glucose production was due to a glycogenolysis flux of around 200–400 µmol per gram of liver per day (Fig. 4j), which agrees well with in vivo estimates during early fasting (Supplementary Table 4). To confirm this observation, we treated liver slices with the glycogen phosphorylase inhibitor CP-91149, which has been shown to reduce hepatic glucose production in mice^47^. Reassuringly, CP-91149 at clinically relevant concentrations strongly reduced glucose production in liver slices (Fig. 4k), confirming that glycogen breakdown is the major source of glucose in these conditions. To investigate if lack of gluconeogenesis was due to insufficient gluconeogenic precursors, we performed experiments with lactate added to the medium. Lactate did increase net glucose production (Fig. 4l). Follow-up 3-^13^C-lactate tracing experiments revealed that lactate carbon entered the tricarboxylic acid cycle and the gluconeogenesis pathway, evidenced by ^13^C-labelled malate, glutamate and glyceraldehyde-3-phosphate (Fig. 4m). Interestingly, the glyceraldehyde-3-phosphate ^13^C_1_ fraction was much higher than malate ^13^C_1_, indicating isotope exchange through pyruvate kinase, which may be near equilibrium in this setting. However, glucose ^13^C labelling remained low (Fig. 4m), suggesting low activity at the fructose 1,6-bisphosphatase step.

Given estimates of metabolic fluxes in liver tissue, it becomes possible to determine system properties such as respiration and carbon flow. With the available uptake/release data, the model estimated an oxygen consumption rate of around 1,000 µmol per gram of liver per day (Extended Data Fig. 4h), which is remarkably close to estimates from human whole-body oxygen consumption, but about half of that measured in rat liver slices (Extended Data Fig. 4h and Supplementary Table 4). Similarly, nutrient uptake rates in rat liver slices were at least two-fold higher than in human slices (Extended Data Fig. 4i), consistent with more rapid metabolism. It has long been appreciated that cellular respiration decreases with body size^48^, which implies that energy metabolism is markedly different between rodents and humans, with possible implications for translating metabolism research from animal models. From the estimated fluxes, we also estimated the carbon flow through liver tissue by tracing the fate of each model substrate to each released product (Extended Data Fig. 4j). This revealed that about half of all carbon metabolized was oxidized to CO_2_, indicating that energy demand drives substrate usage to a large extent. Glucose production required 15–20% of the total carbon, while protein synthesis accounted for another 12–13%. Glycogen was the major carbon source in these conditions and accounted for most of the glucose production. These values are only approximations as our model of liver metabolism is incomplete; nevertheless, they give some insight into the major metabolic routes.

### Liver tissue response to postprandial nutrients and insulin

**Fig. 5:**
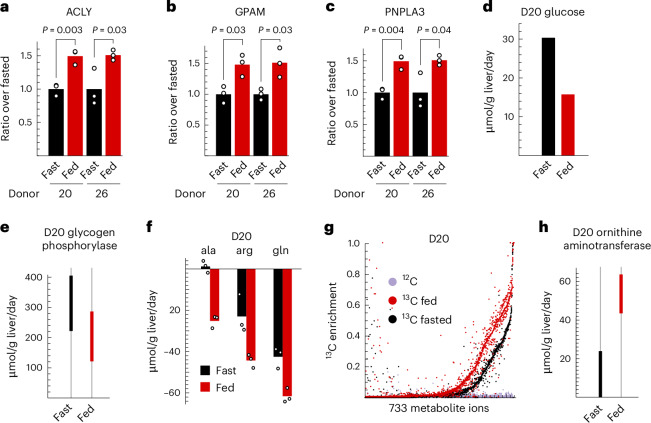
Metabolic response of liver tissue to nutrients and insulin. **a**–**c**, Expression level of mRNAs for ATP-citrate lyase (ACLY; **a**), glycerol-3-phosphate acetyltransferase (GPAM; **b**) and patatin-like phospholipase domain containing 3 (PNPLA3; **c**) in fasted and fed conditions. **d**, Glucose release flux. **e**, 90% confidence intervals for glycogen phosphorylase flux. **f**, Uptake (negative) fluxes for indicated AAs. **g**, Distribution of ^13^C enrichment in the indicated conditions. Dots represent values for individual metabolites, from three tissue slices per condition. **h**, 90% confidence intervals for ornithine aminotransferase flux. Confidence intervals in **e** and **h** were obtained by the profile likelihood method (Methods) based on *n* = 3 independent tissue slices. *P* values were calculated using Student’s two-sided *t*-test from *n* = 3 tissue slices. Source data

**Extended Data Fig. 5:**
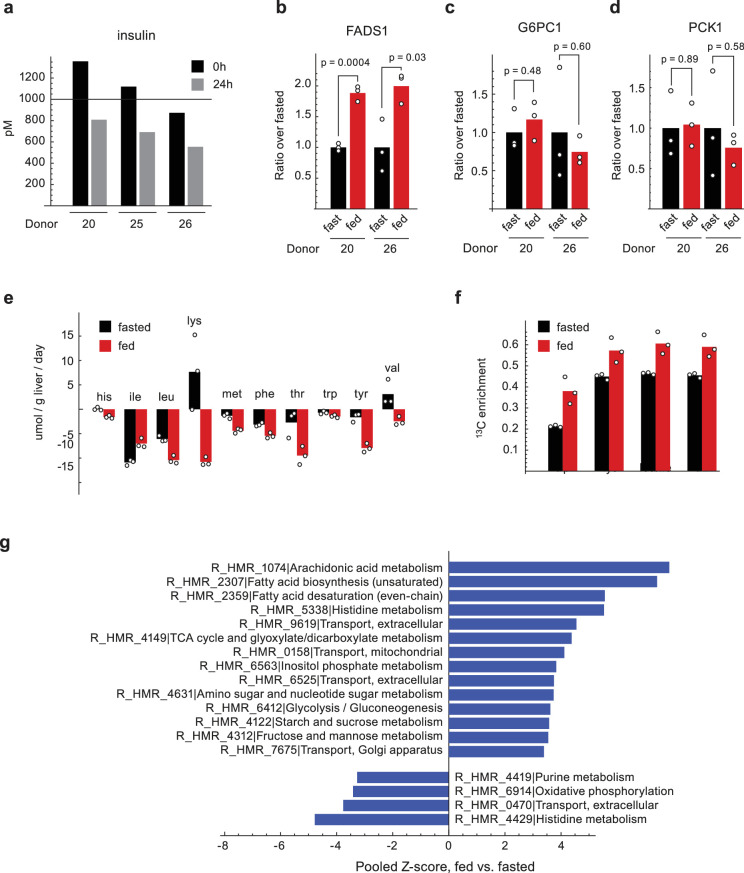
Gene expression and metabolic properties in fasted and fed liver slices. **a**, Insulin concentration in fresh (0 h) and spent (24 h) medium from liver slice cultures. Line indicates nominal fresh medium concentration (1 nM). **b**–**d**, Expression level of mRNAs for fatty acid desaturase 1 (FADS1, b), glucose-6-phosphatase catalytic subunit 1 (G6PC1, c) and phosphoenolpyruvate carboxykinase 1 (PCK1, d) in fasted and fed conditions. **e**, Uptake (negative) and release (positive) rates of essential amino acids in fasted and fed conditions. **f**, ^13^C enrichment in indicated amino acids in fasted and fed conditions. **g**, Reactions from the hepatocyte genome-scale model predicted to be up- or downregulated by insulin based on mRNA expression in fasted and fed liver tissue. p-values in b–d were calculated using Student’s two-sided t-test from n = 3 tissue slices. Data from n = 3 independent tissue slices are shown in e–f. Source data

A major advantage of an ex vivo system is the ability to manipulate the nutrient and hormonal environment in a precise manner. As a case study, we here explored the metabolic response of liver tissue to increased nutrients and insulin, approximating the metabolic environment in the fed (postprandial) state. In presence of 1 nM insulin, similar to human portal vein concentrations^49^, we observed insulin uptake by liver tissue (Extended Data Fig. 5a) and marked increases in known insulin-responsive mRNAs in the de novo fatty acid (Fig. 5a and Extended Data Fig. 5b) and triglyceride (Fig. 5b) synthesis pathways. Interestingly, we also observed induction of *PNPLA3* (Fig. 5c), a major susceptibility gene for fatty liver disease reported to be responsive to insulin and glucose^50^. This suggests that intact human liver tissue is quite insulin sensitive, compared to hepatic cell lines that may require 10–100-fold higher insulin concentrations^51,52^. We did not see suppression of gluconeogenesis mRNAs (Extended Data Fig. 5c, d) that are considered to be more susceptible to insulin resistance^53^, but glucose production was nevertheless reduced in the fed state (Fig. 5d), showing that actual effects on metabolism may not be evident at the mRNA level. MFA indicated that reduced glucose production was mainly due to suppression of glycogenolysis (Fig. 5e and Supplementary Table 8). Uptake of both essential and non-essential AAs increased in the fed state (Fig. 5f and Extended Data Fig. 5e), and liver tissue generally incorporated more ^13^C into metabolic products (Fig. 5g), indicating an overall increase in anabolic metabolism. In particular, essential AA ^13^C enrichment increased (Extended Data Fig. 5f), indicating suppression of endogenous protein catabolism, as previously reported^54,55^. Interestingly, arginine catabolism flux increased in fed conditions (Fig. 5h), suggesting that this pathway is regulated by nutrient state.

## Discussion

In summary, our data demonstrate that human liver tissue cultured ex vivo retains many of the metabolic and physiological characteristics of human liver in vivo. Untargeted isotope tracing in this system yields qualitative data on a wide range of liver pathways, as exemplified by our findings on BCAA transamination and de novo creatine synthesis, while MFA can be used to quantify fluxes for specific pathways of interest. Such direct measurements are important to understand liver physiology, and cannot always be predicted from transcriptomics or proteomics data. As an illustration, an analysis of transcriptomics data from our fed/fasted experiment using a recent genome-scale hepatocyte model^56^ highlighted upregulation of fatty acid synthesis by insulin as expected (Extended Data Fig. 5g and Supplementary Table 9), but did not predict effects on glycogen and arginine metabolism observed using MFA (Fig. 5e,h).

Several important limitations must be pointed out. First, the cohort used in this study was quite small (Supplementary Table 1), and most slices used for deep labelling experiments were from male donors, so that some results may not be applicable to female liver. Although cultured tissue slices overall showed good viability and maintained key liver functions, histology analysis occasionally revealed areas of tissue deterioration, and it remains possible that certain regions had poor access to nutrients or oxygen. Our batch culture set-up also does not fully achieve metabolic steady state, which MFA analysis assumes, and estimated fluxes should therefore be considered as approximations and interpreted carefully. More advanced bioreactor systems that provide a continuous flow of medium^22,24^ might improve nutrient delivery and also help achieve metabolic steady state, and merit further study. Moreover, as with any culture model, the medium composition can strongly affect metabolism. While our human serum-containing medium improves on the Williams E formulation by providing protein, triglycerides and albumin-bound fatty acids, concentration of metabolites such as lactate and urea may affect liver metabolism and should be further investigated. Finally, liver slices naturally cannot fully replicate the intact organ, as nerve connections are severed and organ-to-organ communication is absent, which may impact metabolic processes. In particular, while we find near-physiological levels of glucose production from glycogen in liver slices, gluconeogenesis was very low, possibly because liver glycogen was not fully depleted, or due to lack of hormonal stimulation.

Given the difficulties of measuring metabolism in humans in vivo and the considerable metabolic differences between humans and rodents, we envision that the proposed ex vivo model can be important for translating metabolism research from model organisms to humans. As we show, cultured liver tissue responds to hormones at physiological concentrations and can easily be manipulated by drugs, while comparisons can be made between slices within each patient to minimize variation and examine each individual’s response to treatment. These qualities make ex vivo systems of outstanding interest for metabolism research and pharmaceutical testing. Naturally, these concepts can be extended to any tissue of interest that can be maintained in good condition in culture.

## Methods

### Participants

The study was approved by the Swedish Ethical Review Authority (2020-03841, 2021-05087, 2022-06272-02) and conducted in accordance with ethical guidelines of the 2013 Declaration of Helsinki. Informed written consent was obtained from all participants. There was no compensation for participants. Individuals undergoing hepatectomies due to hepatocellular carcinoma or liver metastasis at the Department of Surgery, Linköping University Hospital were recruited consecutively to the study, with age < 18 years as the sole exclusion criteria. Participants were fasted overnight (no solid food) for at least 8 h but received a preoperative liquid carbohydrate loading of 100.8 g in the evening and 50.4 g in the morning (3 h before surgery). Overall, 45% of all donors of material to the study were female. We did not attempt to analyse sex differences due to the small sample size.

### Quantification of glucose, lactate, triglycerides and insulin

In total, 5 ml of blood was collected in sodium heparin tubes (BD Vacutainer Sodium-Heparin Plasma Tubes, BD Diagnostics). Blood samples were centrifuged at 2,000*g* for 10 min at room temperature. The plasma fraction was aliquoted and stored in −80 °C until analysis. Quantification of glucose, lactate and triglycerides in the plasma samples and cell culture medium, as well as insulin in cell culture medium, was performed by Clinical Chemistry, Laboratory Medicine at Linköping University Hospital, according to their routine analysis.

### Synthesis of culture medium

Williams E medium was synthesized in-house from individual components, according to the formulation given in Supplementary Table 2. For the ^13^C deep labelling medium, all AAs and glucose were replaced with the following ^13^C-labelled counterparts (Cambridge Isotope Laboratories): ^13^C_3_-alanine (CLM-2184-H), ^13^C^6^-glucose (CLM-1396-0), ^13^C_6_-arginine (CLM-2265-H), ^13^C_4_-asparagine (CLM-8699-H), ^13^C_4_-aspartic acid (CLM-1801-H), ^13^C_3_-cysteine (CLM-4320-H), ^13^C_5_-glutamic acid (CLM-1800-H), ^13^C_5_-glutamine (CLM-1822-H), ^13^C_2_-glycine (CLM-1017-0), ^13^C_6_-histidine:HCl:H_2_O (CLM-2264-0), ^13^C_6_-isoleucine (CLM-2248-H), ^13^C_6_-leucine (CLM-2262-H), ^13^C_6_-lysine:2HCl (CLM-2247-H), ^13^C_5_-methionine (CLM-893-H), ^13^C_9_-phenylalanine (CLM-2250-H), ^13^C_5_-proline (CLM-2260-H), ^13^C_3_-serine (CLM-1574-H), ^13^C_4_-threonine (CLM-2261-0), ^13^C_11_-tryptophan (CLM-4290-H), ^13^C_9_-tyrosine (CLM-2263-H) and ^13^C_5_-valine (CLM-2249-H). For the various single isotope tracing experiments, Williams E media were synthesized with ^13^C_6_-leucine (CLM-2262-H), ^13^C_6_-arginine (CLM-2265-H) or 3-^13^C-lactate (CLM-1578-PK). Three liver slices were used for all experiments. Unlabelled (^12^C) Williams E medium (unlabelled nutrients from Merck) was used as control. For cultures containing serum, frozen human serum was obtained from blood banks at Uppsala University Hospital and Karolinska University Hospital, Stockholm, thawed and dialysed in SnakeSkin 10,000 molecular-weight cut-off dialysis tubing (Thermo Fisher Scientific, 88245) to remove small molecules. Williams E medium was then prepared as above but with all concentrations doubled, and mixed at a 50:50 (vol/vol) ratio with dialysed serum to obtain final concentrations of small metabolites equal to those in serum-free medium. Concentrations in dialysed serum were measured by isotope dilution mass spectrometry (Supplementary Table 4) as described below, based on the known concentrations of the ^13^C-labelled nutrients in the synthesized Williams E medium. All media were supplemented with 1% penicillin–streptomycin (PeSt; Gibco,15140148).

### Preparation and culture conditions of liver slices

Human liver tissue and a venous blood sample were obtained from individuals undergoing hepatectomies due to hepatocellular carcinoma or liver metastasis at Linköping University Hospital. Tissue samples (~1 cm^3^) were taken from healthy liver tissue included in the tumour resection, and immediately placed in ice-cold preservation solution (Storeprotect Plus, Carnamedica) and processed within 2 h. Small tissue pieces (~2 mm^3^) were cut out, washed in ice-cold PBS (pH 7.4, Gibco, 10010056) and either snap frozen in a dry-ice/ethanol bath and stored at −80 °C until analysis, or placed in 4% paraformaldehyde (Histolab, 2176) for histopathology. The remaining tissue was cut into slices using a vibratome Leica VT1200S (Leica Biosystems; speed: 0.3–0.6 mm s^−1^, amplitude: 3 mm, step size: 150 or 250 μm) while submerged in ice-cold preservation solution. The resulting tissue slices were punched with a disposable 8-mm biopsy punch (Kai medical, BP-80F) to obtain slices with a consistent size, transferred onto a cell culture insert (hydrophilic PTFE, 0.4 µm, Millicell, Millipore, PICM03050) in six-well plates containing 1.3 ml prewarmed custom-synthesized medium and with or without 50% dialysed human serum and immediately incubated at 37 °C and 5% CO_2_. After a 2 h preincubation, the cell culture inserts were transferred to new six-well plates containing fresh prewarmed medium and returned to the incubator for an additional 22 h. Fresh or cultured liver slices were washed in ice-cold PBS, transferred to microcentrifuge tubes, snap frozen and stored at −80 °C. Spent culture medium was collected, snap frozen and stored in −80 °C.

### Culture of primary human hepatocytes

Primary human hepatocytes were prepared from human liver as previously described^57^ and kept in cryostorage until use. Cells were thawed in DMEM and plated in 24-well plates in attachment medium (DMEM, 5% FBS, 4 μg ml^−1^ insulin, 1 μM dexamethasone) at 300,000 cells and 500 μl medium per well, and incubated for 3 h at 37 °C and 5% CO_2_. Medium was then changed to the custom-synthesized labelled (^13^C) Williams E medium described above (500 μl per well), with the addition of selenium (5 ng ml^−1^), transferrin (5.5 μg ml^−1^), insulin (0.58 ng ml^−1^), dexamethasone (0.1 μM), penicillin (100 U ml^−1^) and streptomycin (100 μg ml^−1^). After 24 h of incubation, 400 μl of medium (supernatant) was collected from each well; cells were then washed twice with 500 μl cold PBS and extracted with cold methanol (500 μl per well). Medium and extracts from two wells were pooled for each replicate and used for LC–MS analysis.

### Rat liver slices

Male Wistar rats (Charles River, Germany) were group-housed in a controlled environment (21 °C, humidity-controlled, reverse 12 h light–dark cycle) with free access to food pellets and tap water for 4 months before euthanasia (deeply anesthetized with isoflurane and decapitated). The liver of one male rat was quickly removed and placed in ice-cold preservation solution and processed in same way as human samples. All procedures were conducted in accordance with the European Union Directive 2010/63/EU, and the protocol was approved by the Ethics Committee for Animal Care and Use at Linköping University.

### ATP content assay

ATP content was determined using an enzymatic assay, normalized to the total protein content of each slice. Frozen tissue slices were homogenized in 200 µl ice-cold 70% ethanol (vol/vol) containing 2 mM EDTA (pH 10.9) using a TissueLyser II (Qiagen) with a 5-mm stainless-steel bead for 3 min at 50 Hz. The homogenate was centrifuged for 10 min at 14,000*g* in 4 °C; the resulting supernatant was used for the ATP assay, and the pellet for protein analysis. The supernatant was diluted 1,000-fold in 0.1 M Tris-HCl containing 2 mM EDTA (pH 7.75). ATP content was measured using a luminescent assay (BioThema, ATP kit SL) in white 96-well plates in a multimode plate reader (Glomax Explorer, Promega) with an external ATP-calibration curve. The pellet was air-dried and reconstituted in 200 µl 1 M sodium hydroxide for 60 min at 50 °C with occasional vortexing. After dilution with water to a concentration of 0.1 M sodium hydroxide, the protein content was determined using a Pierce BCA Protein Assay kit (Thermo Scientific) in clear 96-well plates in a VersaMax microplate reader (Molecular Devices) with an external bovine serum albumin calibration curve.

### Paraffin sectioning and histology

Liver slices cultured for 24 h and fresh liver tissue from four individuals were fixed in 4% buffered paraformaldehyde solution (Histolab, 2176) at 4 °C for 24 h and 48 h, respectively, washed in PBS (Gibco, 10010056) and stored in 70% ethanol at 4 °C before dehydration. Liver tissue was dehydrated in an automatic tissue processor (Leica TP1020), embedded in paraffin (Leica EG 1150 embedding station), cut at a thickness of 4 µm (Thermo Fisher Microm HM355S) and stained with H&E (Histolab, 01820; Leica ST4020 Small Linear Stainer). Tissue viability and morphology (including ballooning, inflammation, steatosis, fibrosis and infiltration of tumour cells) was evaluated by a specialist pathologist.

### Albumin, apolipoprotein B and urea assays

Medium from three tissue slices cultured for 24 h (spent medium) and/or plasma samples from five patients were collected and analysed in duplicate in each assay. Secreted albumin was measured using human albumin ELISA (Thermo Fisher Scientific, EHALB, diluted at 1:50 and 1:1,000,000). Apolipoprotein B was quantified by using the human APOB ELISA kit (R&D Systems, DAPB00, undiluted and diluted at 1:1,000). Urea levels were determined by a colorimetric urea assay (Sigma-Aldrich, MAK006, diluted at 1:15 and 1:25). All three assays were performed according to the manufacturer’s instructions. Data were normalized to weight of tissue slice and hours of incubation.

### TAG assay

TAGs secreted from liver slices were measured using a colorimetric assay (Cayman Chemical, 10010303, undiluted) according to the manufacturer’s instructions. Absorbance was measured at 540 nm in a VersaMax microplate reader (Molecular Devices). Williams E medium from cultures of incubated liver slices for 22 h (spent medium) from four individuals with two to three biological replicates were collected and samples were run as duplicates or triplicates.

### NEFA assay

Medium concentrations of non-esterified fatty acids (NEFAs) were measured using a NEFA-HR(2) reagent, an enzymatic colorimetric method assay (Wako Chemicals, Neuss, Germany), according to the manufacturer’s instructions.

### RNA-seq

Total RNA was extracted from frozen fresh liver tissue pieces and liver slices cultured for 24 h. Briefly, samples were homogenized in 2 ml microtubes (Eppendorf, 0030108078) containing 1 ml Qiazol lysis reagent (Qiagen, 79306) together with a 5-mm stainless-steel bead (Qiagen, 69989) using a TissueLyzer II (Qiagen) for 2 min at 30 Hz. Then, 200 µl chloroform was added to each sample and mixed vigorously by vortexing for 15 s followed by 3 min incubation at room temperature (RT). Samples were centrifuged at 12,000*g* for 15 min at 4 °C and the upper phase containing RNA was collected. Isolation of total RNA was done on a QIAcube liquid handling system (Qiagen) using RNeasy Mini Kit (Qiagen, 74104) and RNase-Free DNase Set (Qiagen, 79254) according to the manufacturer’s instructions. RNA yield and A260:A280 ratio was determined using a NanoDrop 2000 (Thermo Scientific). RNA-seq for the fresh versus cultured experiment was performed at Vienna Biocenter Core Facilities (Vienna, Austria), and reads were mapped to the human genome version hg38. Mapped reads were summarized into counts per Ensembl gene (ENSG) using subread v.2.0.3 and normalized across samples using the DESeq method^58^. RNA-seq for the fed versus fasted experiment was performed by Novogene according to standard protocols, and data were analysed using the Novogene bioinformatics pipeline.

### LC–MS

#### Polar metabolite LC–MS analysis

Media and plasma samples were thawed on ice, and 20 µl of sample was combined with 80 µl of 50:50 HPLC-grade methanol:acetonitrile extraction solvent. Media and plasma samples were then vortexed and left at −80 C° for 30 min to precipitate proteins. To account for variation in tissue mass, tissue samples were weighted and 20 µl of ice-cold extraction solvent made of 40:40:20 HPLC-grade methanol:acetonitrile:water was added per milligram of tissue. Tissue samples were then homogenized with ~300 mg 1 mm zirconium beads using three 10-s cycles at 6,400 Hz, and left at −20 °C for 30 min to precipitate proteins. Samples were centrifuged at 13,000*g* at 4 °C for 10 min, and supernatants were transferred to LC–MS vials containing 200 µl glass inserts, and stored at −80 °C until analysis. An injection volume of 2 µl was used. The sample run order was chosen to block all known experimental factors.

Untargeted LC–MS/MS analysis was performed using a Thermo Q Exactive orbitrap mass spectrometer coupled to a Thermo Vanquish UPLC system. Chromatographic separation of metabolites was achieved using a Millipore (SeQuant) Zic-pHILIC 100 × 2.1 mm 5 µm column maintained at 45 °C, with a 6-min linear gradient starting from a ratio of 90:10 to a ratio of 45:55 ACN:20 mM ammonium bicarbonate at pH 9.6. A Thermo Q Exactive orbitrap mass spectrometer was operated in positive and negative ion modes using a heated electrospray ionization source at a resolution of 30,000, 75 ms ion trap time for MS1 and 15,000 resolution, 50 ms ion trap time for MS2 collection. Data were collected over a mass range of 65–975 *m/z*, using a sheath gas flow rate of 40 units, auxillary gas flow rate of 20 units, sweep gas flow rate of 2 units, spray voltage of 3.5 kV, capillary inlet temperature of 275 °C, auxillary gas heater temperature of 350 °C and a S-lens RF level of 45. For MS2 collection, MS1 ions were isolated using a 1.0-*m/z* window and fragmented using a normalized collision energy of 30 eV. Fragmented ions were placed on dynamic exclusion for 5 s before being allowed to be fragmented again.

#### Amphipathic lipid LC–MS analysis

Amphipathic lipids (including fatty acids and their derivatives) from plasma and media samples were extracted and analysed as described previously^59,60^. For extracting bioactive lipids from tissue samples, 20 µl of 80:20 HPLC-grade ethanol:water per milligram of tissue was used. Tissue samples were then homogenized and centrifuged as described above. For each tissue sample, 75 µl of supernatant was transferred to a Axygen V-bottom plate and mixed with 350 µl of water for solid-phase extraction purification and untargeted LC–MS/MS analysis as described previously^59,60^.

#### LC–MS data analysis

Thermo.raw format data files from the polar LC–MS analysis were converted to mzML format using the msconvert program from Proteowizard v.3.0.6485, using the vendor-provided peak picking (centroiding) algorithm. Untargeted peak detection was done with the mzMine3 (ref. ^61^) software, using the ADAP methods^62^ for chromatogram and peak detection, followed by peak alignment using the JoinAligner module. From the resulting LC–MS peak lists, any peak that was not reproducibly detected in all three replicates of at least one of the experimental groups was removed, resulting in 1,315 LC–MS peaks. Naturally occurring isotopomers, in-source fragments and other mass spectrometry artefacts were removed using the NetID^11^ R package based on *m/z* and retention time (co-elution), resulting in 733 LM–CS peaks annotated to putative compounds.

Manual review of chromatograms and compound identity was performed for 69 compounds (Supplementary Table 10). Compound identity was verified by *m/z* and retention time of pure standards, as well as MS2 spectra matching against library spectra from Massbank of North America (MoNA; https://mona.fiehnlab.ucdavis.edu/) using the cosine metric, and also against GNPS^63^. In addition, metabolite identity was supported by presence of expected mass isotopomers in the ^13^C-labelled material. Metabolites were identified manually for the amphiphatic lipid data, and also for a few cases not captured by the automated peak detection.

MI distributions were computed for each putative metabolite by integrating MS1 peaks at the expected retention time and *m/z* based on the carbon number, generating a total of 9,005 mass isotopomers per sample. To prevent ‘colliding’ LC–MS peaks from unrelated compounds to be misinterpreted as MI peaks, we removed any MI peak that appeared in unlabelled tissue extracts with an apparent MI fraction > 0.05 above the expected binomial distribution of naturally occurring ^13^C. MI fractions were corrected for naturally occurring ^13^C where non-negligible using the binomial transform^64^. The ^13^C enrichment *e* was computed by the formula $e={\sum }_{i=0}^{n}i{x}_{i}/n$ where ${x}_{i}$ is the fraction of mass isotopomer *i*.

### Uptake/release measurements

Uptake and release of metabolites by liver tissues was determined over 22 h of culture, following the initial medium change at 2 h. Metabolite concentrations were measured in ‘spent’ medium conditioned by liver slices (${c}_{\rm{spent}}$), and in medium incubated without tissue slices over the same time period to control for spontaneous changes (${c}_{\rm{control}}$), and the difference ${c}_{\rm{spent}}\mbox{--}{c}_{\rm{control}}$ was taken as an estimate of average uptake/release rates during the 22-h time period. We used three different methods to estimate concentrations. First, for metabolites present in fresh medium, the difference was computed from the known medium concentration $c$ and fold change in peak area $f$ as $\Delta c=c(1\mbox{--}f)$. Second, to verify peak area-derived estimates, we performed absolute quantification in spent medium from ^13^C tracing experiments by the isotope dilution method, mixing samples at a 1:1 ratio with fresh ^12^C medium containing metabolites at known concentrations. Consider a metabolite with measured MID vector $x$ and unknown concentration ${{c}}$ in the spent medium, MID ${x}^{0}$ and known concentration ${c}_{0}$ in the fresh ^12^C medium, and measured MID ${x}^{\rm{mix}}$ in the 1:1 mixture. It then holds that

$$\frac{1}{2}(c+{c}_{0}){x}^{\rm{mix}}=\frac{1}{2}({c\; x}+{c}_{0}{x}^{0})$$

from which we solve for the unknown concentration ${{c}}$, assuming ${x}^{0}$ to be binomial ${\rm{Bin}}(n,0.0107)$. Third, for glucose, lactate and triglycerides, concentrations in fresh and spent medium were measured by accredited metabolite assays performed at the clinical chemistry unit of Linköping University Hospital.

### Model-based ^13^C flux analysis

MFA was performed based on MI fractions measured in 24-h cultured tissue samples and spent medium, assuming metabolic and isotopic steady state. An atom-level model of central liver metabolism was developed iteratively by attempting to fit measured MI fractions and uptake/release fluxes, identifying causes of poor fitting, adjusting the model and repeating until an acceptable fit was found. This analysis assumes isotopic steady state, which is never fully realized in batch cultures; therefore, derived flux estimates should be viewed as approximations. To estimate exchange fluxes between tissue and medium, a pseudo-steady-state model was used where metabolites in spent culture medium were considered as a linear mixture of fresh medium and intracellular metabolites released into the medium. The full model is provided in OpenFLUX format in Supplementary Data 2; schematics in graphML and PDF format are available at https://github.com/Nilsson-Lab-KI/liver-flux-models/, which also tracks the model development history. A more detailed model description is found in Supplementary Note 1.

Model simulation was performed using the EMU framework^13^ implemented in Mathematica v.11 as previously described^65^. Metabolites present in multiple cellular compartments (cytosol, mitochondria, lysosomes or endoplasmic reticulum) were modelled as linear mixtures. The mean and standard deviation of measured MIDs across replicate slices was used for fitting, with standard deviation estimates less than 0.03 set to 0.03 throughout to account for errors not observable in replicates. In addition to uptake/release measurements as described above, literature data for O_2_ consumption, redox demand and protein synthesis and degradation rates were included; see Supplementary Data 3 for a complete description.

The nonlinear model-fitting problem was formulated with MI fractions and fluxes as free variables^66^ and solved using the GAMS modelling framework with the CONOPT solver (GAMS Software) as previously described^65^. Simulations and model solutions were also confirmed independently using the OpenFLUX software^67^. In each case, the best solution from 10 separate optimizations were taken as the optimal flux vector. Goodness of fit was judged using the ${{{\chi }}}^{2}$ statistic with *n* = 149 independent measurements (113 MI fractions from 21 metabolites plus 36 uptake/release fluxes) and *p* = 69 free model parameters (free fluxes and mixture coefficients for compartments), resulting in a one-sided rejection region of ${{{\chi }}}^{2} > 96.6$ (90% quantile). The influence of each individual measurement on flux estimation was assessed from the ${{{\chi }}}^{2}$ residuals (Extended Data Fig. 4b). Flux confidence intervals were computed using the profile likelihood method^68^, by maximizing or minimizing net flux through each reaction.

Carbon flow was computed from the optimal flux vector for each donor as follows. For each substrate $i$, the enrichment ${e}_{{ij}}$ in each internal metabolite $j$ was computed by simulating MIDs at the optimal flux vector with substrate *i* fully labelled and all other substrates unlabelled. For each metabolite $j$ released from the network with release rate ${r}_{j}$, the carbon flow from substrate *i* to metabolite $j$ is then the product ${e}_{{ij}}\,{r}_{j}$. For metabolites that were both taken up and released, we set ${e}_{{ij}}=0$ to discount exchange fluxes.

### Genome-scale metabolic model analysis

The iHepatocytes 2322 model^56^ containing metabolic network structure and reaction–gene associations was downloaded in SBML format from https://metabolicatlas.org/. *z*-scores contrasting the fed and fasted liver slices were computed from the transcriptomics data for each individual and gene, and averaged across the two donors. Pooled *z*-scores for each reaction in the metabolic network were computed by summing the *z*-scores over all associated genes, as previously described^69^. In cases where multiple reactions mapped to the same set of genes, one representative reaction was picked arbitrarily. *z*-scores greater than three were considered significant.

### Data handling and statistics

Student’s two-sided unpaired *t*-test with unequal variances was used for all *P* values presented, computed with Mathematica v.11 (Wolfram Research). *P* values were not corrected for multiplicity. Data distribution was assumed to be normal, but this was not formally tested. Gene Ontology enrichment analysis (Extended Data Fig. 1d) was performed using the MGSA method^70^ and the resulting model-based posterior probabilities and associated standard errors are shown.

Sample size calculation could not be performed since no previous data were available on expected measurement variability in our system. Since analysis of the isotope tracing data from the five donors included here showed good agreement between donors, this sample size was deemed sufficient. Investigators were blinded to donor characteristics during sample allocation, as donor information was not available at the time of allocating donor tissue to specific experiments. Data analysis was not performed blind to the conditions of the experiments. Mass spectrometry data from one donor that was part of an initial pilot experiment were excluded from the final list of five donors due to systematic differences between batches, likely caused by instrument drift. As we did not have sufficient material to perform all assays on tissue from each donor, tissue slices were randomly used for the various assays, as far as available material allowed.

### Reporting summary

Further information on research design is available in the Nature Portfolio Reporting Summary linked to this article.

## Supplementary information

Supplementary Information Supplementary Note 1

Reporting Summary

Supplementary Tables 1–10 Table 1: Baseline characteristics of study cohort. Table 2: Medium formulations. Table 3: Histology data summary. Table 4: Literature-derived flux values. Table 5: Measured serum concentrations. Table 6: Substrate ^13^C enrichment. Table 7: Reporter metabolite list. Table 8: Confidence intervals on metabolic fluxes. Table 9: Transcriptomics analysis with the iHepatocytes model. Table 10: LC–MS peak annotation summary.

Supplementary Data 1 LC–MS dataset.

Supplementary Data 2 MFA model in OpenFLUX format.

Supplementary Data 3 MID and flux data used for MFA model fitting.

## Source data

Source Data Fig. 1 Statistical source data.

Source Data Fig. 2 Statistical source data.

Source Data Fig. 3 Statistical source data.

Source Data Fig. 4 Statistical source data.

Source Data Fig. 5 Statistical source data.

Source Data Extended Data Fig. 1 Statistical source data.

Source Data Extended Data Fig. 2 Statistical source data.

Source Data Extended Data Fig. 3 Statistical source data.

Source Data Extended Data Fig. 4 Statistical source data.

Source Data Extended Data Fig. 5 Statistical source data.

## Data Availability

The LC–MS dataset is available at MetaboLights under accession no. MTBLS10481. RNA-seq data are available at the NCBI Gene Expression Omnibus under accession no. GSE271041. The MFA model and associated data are available in the Supplementary Information. Source data are provided with this paper.

## Code availability

Mathematical code for the MFA is available at https://github.com/Nilsson-Lab-KI/liver-flux-analysis/.
