# Supplementary material for: Global 13C tracing and metabolic flux analysis of intact human liver tissue ex vivo
Source: Nat Metab. 2024 Aug 29;6(10):1963–75. doi: 10.1038/s42255-024-01119-3 (PMC11496108; doi:10.1038/s42255-024-01119-3)
Supplement: Supplementary file 1 — Supplementary Note 1 [file 42255_2024_1119_MOESM1_ESM.pdf]

nature metabolism

Supplementary information https://doi.org/10.1038/s42255-024-01119-3

Global ^{13}C tracing and metabolic flux analysis of intact human liver tissue ex vivo

In the format provided by the authors and unedited

Supplementary Note 1

This supplementary note provides additional documentation on the metabolic flux analysis.

Metabolic network model

The full metabolic network mode is available on github at

https://github.com/Nilsson-Lab-KI/liver-flux-models

To ease navigating the model, the github repository includes a schematic graphML and PDF formats. Note that in this schematic, nodes with large number of inputs such as glutamate (`glu-L`) have been duplicated to allow for a legible network layout.

Amino acids

We included metabolism of amino acids ala, arg, asp, gln, glu, gly and ser, as these had substantial uptake/release fluxes and generate distinctive MIDs. To represent contribution to amino acids from endogenous protein catabolism, we included a lysosomal amino acid compartment.

Mitochondria

To allow for exchange of reducing equivalents into the mitochondrion, we included the malate-aspartate shuttle. A model of the respiratory chain was included to allow estimating oxygen consumption at complex IV.

Sugar metabolism

We includes glycolysis, the pentose phosphate pathway, glycogen metabolism and glucose phosphatase (`G6PPer`) to model hepatic glucose production. As glucose phosphatase is thought to be located in the endoplasmic reticulum [1], we included this compartment in the model. Consequently, glucose (`glc`) and glucose-6-phosphate (`g6p`) occur in both the cytosol and ER compartments in the model.

Triacylglycerols, fatty acids and glycerol

Although we did not measure fatty acid oxidation directly, we included the mitochondrial fatty acid oxidation pathway in the model to account for the possibility of acetyl-CoA carbon deriving from fatty acids. We included glycerol synthesis from trioses (`G3PD1`, `GLYCPH`) to represent demand for the glycerol backbone for triacylglycerol synthesis, which was constrained by measurements of VLDL production. We did not explicitly represent triacylglycerol metabolism.

Energy expenditure

The demand for energy (ATP) and reducing equivalents (NADPH) was modeled by artificial reactions **`ATPASES`** and **`NADPH_REDOX`**, respectively. These were constrained to a rather wide range based on literature values (see below). However, since the model also allows for futile cycles such as phosphofructokinase–fructose bisphosphatase (**`PFK`**, **`FBP`**) which can consume ATP and are difficult to estimate from MID data, flux through these demand reactions should be interpreted carefully.

Measured fluxes

Measured net uptake/release fluxes used to fit the model are listed in Supplementary data 3. In addition to experimentally measured values, rough estimates of ATP and NADPH demand (**`atpases`**, **`nadph_demand`**), oxygen consumption (**`o2`**) and protein synthesis and degradation rates (**`protsyn_`**, **`protdeg_`**) were taken from literature [2–4]. Large standard deviations were set for these values to limit their influence on the model fit.

To estimate exchange fluxes between tissue and medium from observed MIDs, a pseudo-steady-state model was used where the MID of a metabolite in spent culture medium was modeled as a linear mixture of contribution from fresh medium and from intracellular metabolites released into the medium, as depicted below.

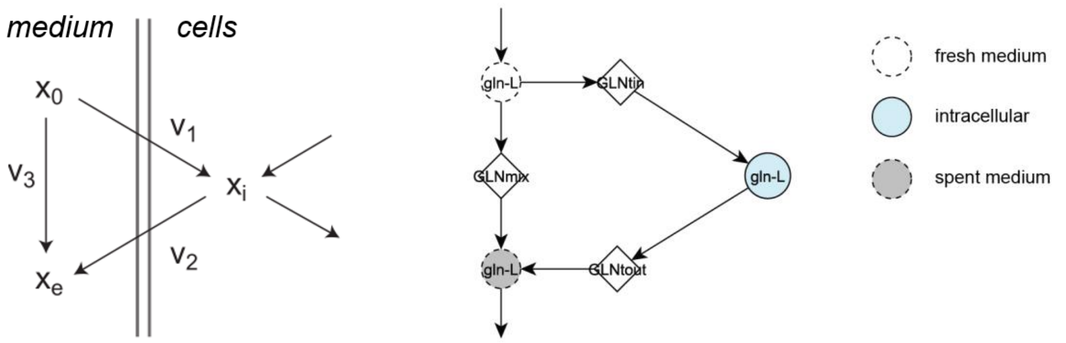

Model of medium exchange fluxes. Left, schematic indicating metabolite MIDs x and fluxes v. Right, example representation of glutamine exchange in the MFA model, as shown in the graphML file.

Here x_0 is the fresh medium MID for a labeled substrate, x_i is the observed intracellular MID, and x_e is the "extracellular" MID observed in spent medium at the end of the culture. Fluxes v_1 and v_2 are the absolute (directional) uptake and release fluxes, such that $v_2 - v_1$ is the net release flux. The flux v_3 is artificial, representing the proportion of the medium substrate that is not metabolized by cells. The MID x_0 is known, x_i and x_e are measurable, and the sum $v_1 + v_3$ is fixed by the total amount of substrate in the medium. Hence, the free parameters in this system are v_1 and v_2, which can be estimated by fitting the system.

Elementary metabolite unit (EMU) model

Model simulation and fitting was performed using the Elementary metabolite unit (EMU) framework [5]. Rotational symmetry in fumarate and succinate were handled using EMU equivalence classes.

Labeled substrates were modeled with 99% purity, while unlabeled substrates were modeled as a natural (binomial) MID with 1.07% ^{13}C, so that measured MIDs could be fitted directly without correction for natural ^{13}C abundance. For metabolites that exist in more than one compartment, a linear mixture model was used to fit the measured MID, with the mixture coefficients as free parameters.

Code availability

Source code for the metabolic flux analysis is available as a Mathematica notebook at

https://github.com/Nilsson-Lab-KI/liver-flux-analysis

Running this notebook requires the GAMS optimization framework and the CONOPT nonlinear optimization solver. All code was tested with Mathematica v.11 (Wolfram Research).

Notes on interpretation of estimated fluxes

The confidence intervals reported reflect for each reaction the range of values of net flux that are compatible with the measurement data. Because the underlying network model is quite complex and considers a wide variety of possible metabolic states, these intervals are often quite wide, and particularly for reactions where the available data is not informative for estimating flux. However, even wide intervals can be useful to indicate whether a reaction must be active (when the confidence interval excludes zero) and to determine the direction of net flux. It must be noted that each endpoint of each confidence interval corresponds to a distinct flux state, so that it is not possible to combine values across reactions, for example to draw conclusions about metabolic ratios; instead, a new confidence interval must be computed for the quantity of interest.

One reason for wide confidence intervals is the existence of metabolic cycles, such as the futile cycle formed by phosphofructokinase and fructose bisphosphatase. For reactions that can participate in such cycles, confidence intervals can be very wide, since isotope labeling data is often not informative in this case. Metabolic cycles can also involve multiple steps: one example is the cycle PEPCK → pyruvate kinase → pyruvate carboxylase → oxaloacetate transfer to the cytosol → PEPCK which can be seen to affect the PEPCK confidence interval. Because of such cycles, one must be careful when interpreting flux through an enzyme in terms of pathway flux. For example, in our analysis the upper bound for phosphofructokinase (**`FPK`**) is very large, but this does not indicate that net glucolytic flux is of this magnitude. In this case, the glyceraldehyde 3-phosphate dehydrogenase (**`GAPD`**) reaction is a better

indicator of glycolytic flux, as it is not affected by futile cycles, and consequently its confidence interval is much smaller (Suppl Table 8).

References

1. Kalant, N., Parniak, M. & Lemieux, M. Compartmentation of glucose 6-phosphate in hepatocytes. **248**, 5 (1987).

2. Lewis, C. A. *et al.* Tracing Compartmentalized NADPH Metabolism in the Cytosol and Mitochondria of Mammalian Cells. *Molecular Cell* **55**, 253–263 (2014).

3. Rolfe, D. F. & Brown, G. C. Cellular energy utilization and molecular origin of standard metabolic rate in mammals. *Physiological reviews* **77**, 731–758 (1997).

4. Booth, J., Boyland, E. & Cooling, C. The respiration of human liver tissue. *Biochemical Pharmacology* **16**, 721–724 (1967).

5. Antoniewicz, M. R., Kelleher, J. K. & Stephanopoulos, G. Elementary metabolite units (EMU): a novel framework for modeling isotopic distributions. *Metabolic engineering* **9**, 68–86 (2007).
