# Supplementary material for: Global 13C tracing and metabolic flux analysis of intact human liver tissue ex vivo
Source: Nat Metab. 2024 Aug 29;6(10):1963–75. doi: 10.1038/s42255-024-01119-3 (PMC11496108; doi:10.1038/s42255-024-01119-3)
Supplement: Supplementary file 2 — Reporting Summary [file 42255_2024_1119_MOESM2_ESM.pdf]

nature portfolio

Corresponding author(s): Roland Nilsson

Last updated by author(s): Jun 20, 2023

Reporting Summary

Nature Portfolio wishes to improve the reproducibility of the work that we publish. This form provides structure for consistency and transparency in reporting. For further information on Nature Portfolio policies, see our Editorial Policies and the Editorial Policy Checklist.

Statistics

For all statistical analyses, confirm that the following items are present in the figure legend, table legend, main text, or Methods section.

n/a	Confirmed	
☐	☒	The exact sample size (*n*) for each experimental group/condition, given as a discrete number and unit of measurement
☐	☒	A statement on whether measurements were taken from distinct samples or whether the same sample was measured repeatedly
☐	☒	The statistical test(s) used AND whether they are one- or two-sided<br>*Only common tests should be described solely by name; describe more complex techniques in the Methods section.*
☒	☐	A description of all covariates tested
☒	☐	A description of any assumptions or corrections, such as tests of normality and adjustment for multiple comparisons
☐	☒	A full description of the statistical parameters including central tendency (e.g. means) or other basic estimates (e.g. regression coefficient) AND variation (e.g. standard deviation) or associated estimates of uncertainty (e.g. confidence intervals)
☐	☒	For null hypothesis testing, the test statistic (e.g. *F*, *t*, *r*) with confidence intervals, effect sizes, degrees of freedom and *P* value noted<br>*Give P values as exact values whenever suitable.*
☒	☐	For Bayesian analysis, information on the choice of priors and Markov chain Monte Carlo settings
☒	☐	For hierarchical and complex designs, identification of the appropriate level for tests and full reporting of outcomes
☒	☐	Estimates of effect sizes (e.g. Cohen's *d*, Pearson's *r*), indicating how they were calculated

Our web collection on statistics for biologists contains articles on many of the points above.

Software and code

Policy information about availability of computer code

Data collection	No software was used
Data analysis	Mathematica code for the metabolic flux analysis is available at https://github.com/Nilsson-Lab-KI/liver-flux-analysis

For manuscripts utilizing custom algorithms or software that are central to the research but not yet described in published literature, software must be made available to editors and reviewers. We strongly encourage code deposition in a community repository (e.g. GitHub). See the Nature Portfolio guidelines for submitting code & software for further information.

Data

Policy information about availability of data

All manuscripts must include a data availability statement. This statement should provide the following information, where applicable:

- Accession codes, unique identifiers, or web links for publicly available datasets
- A description of any restrictions on data availability
- For clinical datasets or third party data, please ensure that the statement adheres to our policy

The LCMS data set is available at Metabolights, accession no. MTBLS10481. The RNA-seq data is available at the NCBI Gene Expression Omnibus, accession no. GSE271041. The metabolic flux analysis model and associated data is available in supplementary information.

Research involving human participants, their data, or biological material

Policy information about studies with human participants or human data. See also policy information about sex, gender (identity/presentation), and sexual orientation and race, ethnicity and racism.

Reporting on sex and gender	We believe findings apply to both sexes, as the overall study was not heavily skewed towards either sex: 13 out of the 29 donors that contributed liver tissue to this study (including follow-up experiments) were female (45%), and 4 out of 8 donors of tissue used for mass spectrometry analysis were female. We did not select donors on basis of sex, and due to the small sample size, we judge that post-hoc tests for differences between sexes are not meaningful. Due to ethical and legal restrictions on disseminating personal information, we would prefer to present data on a group level rather than disclose information about individual donors, and we therefore chose not to indicate the sex of the specific donor used for particular experiments.
Reporting on race, ethnicity, or other socially relevant groupings	No such variables were used in the study.
Population characteristics	The characteristics of the study cohort is provided in Supplementary Table 1.
Recruitment	Patients over 18 years of age undergoing resection of liver tumors at Linköping University were given the possibility to participate in the study. Any patients that consented to participate were included. Notable biases in this cohort is higher age (median 69 years) and higher prevalence of diabetes (32%) compared to the general population. See Supplementary Table 1 for detailed characteristics of the cohort.
Ethics oversight	The study was approved by the Swedish Ethical Review Authority (2020-03841, 2021-05087, 2022-06272-02)

Note that full information on the approval of the study protocol must also be provided in the manuscript.

Field-specific reporting

Please select the one below that is the best fit for your research. If you are not sure, read the appropriate sections before making your selection.

☒ Life sciences ☐ Behavioural & social sciences ☐ Ecological, evolutionary & environmental sciences

For a reference copy of the document with all sections, see nature.com/documents/nr-reporting-summary-flat.pdf

Life sciences study design

All studies must disclose on these points even when the disclosure is negative.

Sample size	Sample size calculation could not be performed since no previous data was available on expected measurement variability in our system. Since analysis of the isotope tracing data from the 5 donors included here showed good agreement between donors, this sample size was deemed sufficient for the present study. We strived for a similar sample size for other assays used, but final sample numbers were also limited by availability of patient material.
Data exclusions	Mass spectrometry data from one donor that was part of an initial pilot experiment was excluded from the final list of 5 donors due to systematic differences between batches, likely caused by instrument drift.
Replication	Findings from the initial group of 5 donors were successfully validated in independent isotope tracing experiments in a second group of donors, as described in the manuscripts. All findings investigated in these follow-up experiments were successfully validated. We did not attempt additional replication beyond that described in the main text.
Randomization	Donors were randomly assigned to experimental group (no serum, human serum; fasted and fed medium compositions). Choice of donor material to be used for mass spectrometry and other molecular assays was likewise random.
Blinding	Investigators were blinded to donor characteristics during sample allocation, as donor information was not available at the time of allocating donor tissue to specific experiments. Data analysis was not performed blind to the conditions of the experiments.

Reporting for specific materials, systems and methods

We require information from authors about some types of materials, experimental systems and methods used in many studies. Here, indicate whether each material, system or method listed is relevant to your study. If you are not sure if a list item applies to your research, read the appropriate section before selecting a response.

Materials & experimental systems

n/a	Involved in the study	
☒	☐	Antibodies
☒	☐	Eukaryotic cell lines
☒	☐	Palaeontology and archaeology
☐	☒	Animals and other organisms
☒	☐	Clinical data
☒	☐	Dual use research of concern
☒	☐	Plants

Methods

n/a	Involved in the study	
☒	☐	ChIP-seq
☒	☐	Flow cytometry
☒	☐	MRI-based neuroimaging

Animals and other research organisms

Policy information about studies involving animals; ARRIVE guidelines recommended for reporting animal research, and Sex and Gender in Research

Laboratory animals	Wistar rat (Charles River, Germany), age 4 months.
Wild animals	No wild animals were used.
Reporting on sex	One male rat was used.
Field-collected samples	No field collected samples.
Ethics oversight	Animal handling was conducted in accordance with the European Union Directive 2010/63/Eu, and the protocol was approved by the Ethics Committee for Animal Care and Use at Linköping University.

Note that full information on the approval of the study protocol must also be provided in the manuscript.

Plants

Seed stocks	*Report on the source of all seed stocks or other plant material used. If applicable, state the seed stock centre and catalogue number. If plant specimens were collected from the field, describe the collection location, date and sampling procedures.*
Novel plant genotypes	*Describe the methods by which all novel plant genotypes were produced. This includes those generated by transgenic approaches, gene editing, chemical/radiation-based mutagenesis and hybridization. For transgenic lines, describe the transformation method, the number of independent lines analyzed and the generation upon which experiments were performed. For gene-edited lines, describe the editor used, the endogenous sequence targeted for editing, the targeting guide RNA sequence (if applicable) and how the editor was applied.*
Authentication	*Describe any authentication procedures for each seed stock used or novel genotype generated. Describe any experiments used to assess the effect of a mutation and, where applicable, how potential secondary effects (e.g. second site T-DNA insertions, mosiacism, off-target gene editing) were examined.*
